# Assessment of biomarkers and clinical parameters as predictors of survival in patients with chagasic heart failure

**DOI:** 10.1371/journal.pntd.0011847

**Published:** 2023-12-18

**Authors:** Edimar Alcides Bocchi, Guilherme Veiga Guimarães, Cristhian Espinoza Romero, Paula Keiko Sato, Vera Lúcia Teixeira de Freitas, Edite Hatsumi Yamashiro Kanashiro, Célia Regina Furuchó, Fatima Das Dores Cruz, Érika Shimoda Nakanishi, Felipe Delatorre Busser, Rita Cristina Bezerra, Elizabeth Visone Nunes Westphalen, Mussya Cisotto Rocha, Maria Aparecida Shikanai Yasuda

**Affiliations:** 1 Heart Failure Clinics, Instituto do Coração Hospital das Clinicas HCFMUSP, Faculdade de Medicina, Universidade de Sao Paulo, Sao Paulo, Brazil; 2 Laboratory of Medical Investigation in Immunology (LIM-48), Hospital das Clinicas HCFMUSP, Faculdade de Medicina, Universidade de Sao Paulo, São Paulo, Brazil; 3 Departament of Infectious Diseases, Faculdade de Medicina FMUSP, Universidade de Sao Paulo, São Paulo, Brazil; 4 Center of Parasitology and Immunology, Instituto Adolfo Lutz, São Paulo, São Paulo, Brazil; 5 Laboratory of Medical Investigation in Parasitology (LIM-46), Hospital das Clinicas HCFMUSP, Faculdade de Medicina, Universidade de Sao Paulo, São Paulo, Brazil; University of North Carolina at Chapel Hill School of Medicine, UNITED STATES

## Abstract

**Background:**

Chagas disease, endemic in Latin America and spreading globally due to emigration, has a significant health burden, particularly in relation to chagasic heart failure (HF). Chagasic cardiomyopathy (CCM) is characterized by chronic inflammatory myocardial disease. This study aimed to identify inflammatory parameters and biomarkers that could aid in the management of patients with chagasic HF.

**Methods and findings:**

A cohort study was conducted at a tertiary cardiology single-center over a mean follow-up period of 2.4 years. The study included patients with HF secondary to CCM enrolled between October 2013 and July 2017. Various clinical parameters, echocardiography findings, parasitemia status, brain natriuretic peptide (BNP) and troponin T (TnT) levels, and inflammatory biomarkers (IL-6, IL-10, IL-12p70, IL-17A, adiponectin, and IFN-γ) were assessed. The study encompassed a cohort of 103 patients, with a median age of 53 years and 70% being male. The left ventricular ejection fraction (LVEF) was 28%, with 40% of patients classified as NYHA II functional class. The median BNP level was 291 pg/ml. The observed mortality rate during the study period was 38.8%. Predictors of lower survival were identified as elevated levels of BNP, TnT, reduced LVEF, and increased adiponectin (thresholds: BNP > 309 pg/ml, TnT > 27.5 ng/ml, LVEF < 25.5%, adiponectin > 38 μg/mL). Notably, there was no evidence indicating a relationship between parasitemia and the inflammatory parameters with lower survival in these patients, including INF-γ, IL-6, IL-10, IL12-(p70), and IL17a.

**Conclusion:**

Despite the presence of a chronic inflammatory process, the evaluated inflammatory biomarkers in this cohort were not predictive of survival in patients with chagasic HF with reduced ejection fraction (HFrEF). However, reduced LVEF, elevated BNP, adiponectin levels, and troponin T were identified as predictors of lower survival in these patients.

## Author summary

Chagas disease, affecting approximately 7 million individuals worldwide, leads to worse outcomes in patients with chagasic cardiomyopathy (CCM) and heart failure with reduced ejection fraction (HFrEF) compared to other etiologies. The chronic inflammatory process associated with Chagas disease is thought to contribute to these adverse outcomes. Inflammatory biomarkers such as TNF-α, IL-6, IL-1β, and angiotensin II are released due to hemodynamic stress in HF and are considered indicators of inflammation. The role of *T*. *cruzi* parasitemia in the progression of chagasic cardiomyopathy remains controversial. Additionally, brain natriuretic peptide (BNP) and cardiac troponin are essential biomarkers for assessing mechanical stress and myocardial injury, respectively, due to their prognostic value. However, studies correlating inflammatory biomarkers with prognosis in patients with CCM have yielded conflicting results. In this study, inflammatory biomarkers were not found to be predictors of survival in patients with HFrEF of chagasic etiology. Instead, reduced left ventricular ejection fraction (LVEF), elevated BNP, and TnT levels were identified as strong predictors of lower survival. This suggests that in patients with advanced chagasic heart failure, the role of inflammatory markers in predicting survival may be limited.

## Introduction

Chagas disease, caused by *Trypanosoma cruzi*, is a prevalent condition affecting approximately 7 million individuals worldwide [1,2]. The disease burden is particularly high in countries like Argentina, Brazil, Mexico, and Bolivia [1–5]. Cases of Chagas disease have also been reported in the United States, Europe, Australia, and Japan [1,2,5]. Despite the availability of conventional heart failure (HF) therapies, patients with chagas cardiomyopathy (CCM) and heart failure with reduced ejection fraction (HFrEF) experience worse quality of life, higher hospitalization rates, and increased mortality compared to other etiologies [1,2]. The underlying chronic inflammatory process in Chagas disease may contribute to these adverse outcomes [6,7].

The inflammation observed in Chagas disease can be both a cause of *T*. *cruzi* infection and a consequence of HF, playing a central role in disease pathogenesis and progression [6,7]. Hemodynamic stress associated with HF triggers the release of proinflammatory cytokines such as TNF-α, IL-6, IL-1β, and angiotensin II, which are considered biomarkers of inflammation [6,7]. Despite numerous studies establishing the association between inflammation measures and HF severity and prognosis, clinical trials of anti-inflammatory therapies have shown limited success [6,7]. It is crucial to include the measurement of other biomarkers, such as brain natriuretic peptide (BNP) and cardiac troponin, as indicators of mechanical stress and myocardial injury, respectively, due to their prognostic value [6,7]. The role of *T*. *cruzi* parasitemia in the progression of chagasic cardiomyopathy remains controversial [8–11].

The objective of this study was to examine the association of biomarkers and parasitemia with the occurrence of the composite primary outcome in patients with stable chronic HFrEF of Chagas’ etiology. By investigating these factors, we aim to enhance our understanding of the disease and its implications for patient management.

## Methods

### Ethics statement

The study complied with the Declaration of Helsinki and the Research Ethics Board of the Heart Institute (SDC: 3394/09/145), the Human Subject Protection Committee at the Clinics Hospital of the University of São Paulo Medical School (CAE 32344314700000068 and CAPPesq 1043/07). All participants provided written informed consent.

### Study design

This was a single-center, cohort study designed to determine the association into biomarkers and parasitemia with the occurrence of the primary outcome in patients with chronic stable HFrEF of Chagas’ etiology during a follow-up period of 5 years. The primary outcome was a composited of death from all-cause mortality and heart transplantation. All-cause mortality and heart transplantation were also analyzed separately. All patients diagnosed with HF secondary to any Chagas cardiomyopathy routinely followed up at our Heart Failure Outpatient Clinic from October 2013 to July 2018 were initially considered for the study.

Patients with positive serology for Chagas disease and left ventricular systolic dysfunction underwent electrocardiogram, echocardiogram and chest X-ray, being classified as cardiomyopathy of the clinical form of Chagas disease. Patients were evaluated for heart failure according to the New York Heart Association (NYHA) classification. All patients were instructed to keep taking their medications throughout the study period.

### Population

We assessed the eligibility of 684 consecutive patients being treated in the heart failure clinic of a tertiary hospital. Inclusion criteria in the screening were Chagas cardiomyopathy, age over 18 years, left ventricular ejection fraction < 50%, and receiving optimized guideline-directed medical therapy (GDMT). The GDMT was considered when they were using at least 50% of recommended dose of angiotensin-converting enzyme inhibitors or angiotensin receptor blockers (ACE-I/ARB), betablockers and spironolactone), without recent hospitalizations in the last 6 weeks. Patients with pulmonary disease, chronic renal disease, peripheral neuropathy, history of stroke, body mass index (BMI) > 30 kg/m^2^, history of smoking, and those with a defibrillator implant were not eligible.

### Echocardiography

The patients underwent a 1-dimensional (M-mode), 2-dimensional (mode B) transthoracic echocardiographic study with pulsed, continuous, and color Doppler. The echocardiogram was performed only once after inclusion. Sequoia 512 equipment (Acuson, Mountain View, CA) was used, with the coupled multifrequency transducer, model 3V2c, of 2.5–4.0 MHz, according to the recommendations of the American Society of Echocardiography and the European Association of Cardiovascular Imaging. Lang cardiac morphological parameters, systolic function indexes, and diastolic function indexes were analyzed.

### Serum biomarkers

Peripheral blood samples were collected once at time of study enrollment. BNP level was measured using a direct chemiluminescence test (Siemens Healthcare Diagnostics, Tarrytown, New York); cytokine production was tested by ELISA for IL-6, IL-10, IL-12p70, IL-17A and IFN-γ (eBioscience, San Diego, CA, USA); Serum TnT analysis was performed using electrochemiluminescence immunoassays on a cobas 8000 e801 module, (Roche Diagnostics, Mannheim, Germany). Galectin was measured using the MILLIPLEX MAP KIT (Merck Laboratories) with Luminex xMAP technology. The serum concentration of adiponectin was tested by an enzyme-linked immunosorbent assay (ELISA) method.

### Parasitemia

Blood culture tube assays were performed after 30, 60, 90 and 120 days. Results are expressed as the number of positive tubes divided by the total number of tubes examined (%positive tubes). The result was considered positive if any tube was positive and negative if all were negative.

### Qualitative and quantitative PCR

Qualitative conventional PCR (cPCR) was performed using S35 and S36 primer pair targeting a 330-bp sequence from kinetoplast DNA of *T*. *cruzi* (Invitrogen, Thermo Fisher Scientific, Carlsbad, CA, USA), as previously described [12] Negative controls were included in each PCR experiment, to monitor for possible contamination. The samples of patients were processed in duplicate, one containing parasite DNA, 2fg of DNA from Y strain were used to verify the presence of inhibitors.

Quantitative PCR (qPCR) employed a nuclear primer pair, TCZ3/TCZ4, which amplifies a 149 bp microsatellite sequence, as previously reported [12] We generated a standard amplification curve with DNA from blood spiked with 8x10^5^ to 8x10^-2^ par Eq/mL. Appropriate negative controls for contamination detection at any stage of the procedure and positive controls were employed to ensure reproducible results in all experiments.

### Statistical analysis

Data are presented as median (range) or mean ± standard deviation (SD), according to data distribution as assessed by the Kruskal-Wallis test. The association between biomarkers of patients diagnosed with Chagas disease and survival was explored by univariate Cox regression analysis and, subsequently, the variables that were significant by multivariate Cox regression. In the univariate analysis, those with p up to 0.10 were considered for the multivariate, and in the multivariate analysis those with p <0.05 were considered significant.

Receiver-operating character (ROC) curves were analyzed according to the sensitivity and specificity of biomarkers (BNP, TnT, and adiponectin) and left ventricular ejection fraction at rest to predict patient mortality risk. The area under the curve (AUC) of 0.9, 0.8 to 0.89, 0.7 to 0.79, 0.6 to 0.69, or < 0.6 were classified as excellent, very good, good, fair, and poor, respectively. The optimal threshold, sensitivities, specificities, positive predictive values, and negative predictive values were calculated by ROC analysis. The best cutoff values for mortality prediction were determined by the maximum of the Youden index (i.e., sensitivity plus specificity minus one) calculated from the ROC analysis. The Hosmer Lemeshow goodness-of-fit test was used to test the calibration of the scoring system.

Kaplan-Meier analysis was used to determine cardiovascular event-free survival. The log-rank test was used to determine the statistical significance of the Kaplan-Meier analyses. All statistical analyses were performed using SPSS software (IBM Corp., Statistics for Windows, version 21.0, NY, USA), with p values < 0.05 considered statistically significant.

## Results

After the screening, 103 of 684 patients with HF due to Chagas disease were included in the study and the others 581 patients were not included because they presented exclusion criteria mentioned above. All patients were under GDMT, including ACE-I or ARB, beta-blockers, and spironolactone, at the maximum tolerated doses. The first inclusion occurred in October 2013, and patients were followed until July 2018 with a mean follow-up of 2.4 ± 1.4 years. Patients were followed-up by phone interviews with the patient and/or family members, and data record hospital. The study event was death due to cardiovascular causes or the need for a heart transplant (status 1). The baseline characteristics of the patients were described in Table 1. Initial sample analysis revealed 35.9% positive parasitaemia through conventional qualitative PCR. When considering all samples, an additional 16 and 5 new positive cases were identified in the second and third samples, respectively. This brought the total number of cases with positive parasitemia to 56.3% (58 out of 103). In terms of quantitative PCR, 15.8% (13 out of 82) of cases tested positive in the first sample. Following three sample collections, 18.4% (16 out of 87) exhibited positive parasitaemia. Univariable Cox regression analyses were conducted to explore the association between mortality and various patient and disease-related variables for the entire cohort. The results showed no significant association between mortality and INF-γ, IL-6, IL-10, IL12-(p70), IL17a, and galectin. However, there was a significant association between mortality and BNP, TnT, adiponectin, and LVEF. Multivariate analysis identified elevated BNP, TnT, and adiponectin as independent predictors of mortality, and lower LVEF was also diagnosed as an independent predictor of mortality (Table 2). There was no relationship between the positive parasitemia with lower survival or lower LVEF.

**Table 1:** Baseline characteristics of population.

Variable	Median, n = 103	Death		p
		No	Yes	
**Age, y**	53 (43–72)	52	53	0.552
**Male (%)**	70 (68)	52 (65.8)	18 (75)	0.399
**White race (%)**	61 (59.2)	47 (59.5)	14 (58.3)	0.936
**Hypertension (%)**	46 (44.7)	37 (46.8)	9 (37.5)	0.420
**Diabetes (%)**	32 (31.1)	23 (29.1)	9 (37.5)	0.437
CAD (%)*	22 (21.4)	19 (24.1)	3 (12.5)	0.227
**Amiodarone (%)**	26 (25.2)	18 (22.8)	8 (33.3)	0.297
**Furosemide (%)**	82 (79.6)	62 (78.5)	20 (83.3)	0.605
GDMT (%)*				
**ACE-I/ARB (%)**	87 (84.4)	66 (83.5)	20 (83.3)	0.981
**Beta-blockers (%)**	92 (89.1)	71 (89.9)	21 (87.5)	0.742
**Spironolactone (%)**	76 (73.8)	59 (74.7)	17 (70.8)	0.707
**Digoxin (%)**	31 (30.1)	23 (29.1)	8 (33.3)	0.693
**NYHA functional class**				<0.001
**II (%)**	42 (40.8)	34 (43)	8 (33.3)	
**III (%)**	21 (20.4)	7 (8.9)	14 (58.3)	
**LVEF (%) n = 101**	28.0 (23–72)	32	24	<0.001
**IFn-y, n = 103**	218.9 (39–1967)	1312.6	1049.8	0.577
**IL6, n = 47**	11.27 (3.0–93)	117.1	45.0	0.101
**IL10, n = 63**	6.0 (3.9–18.6)	26.2	19.1	0.623
**IL12p70, n = 50**	7.95 (3.9–77.4)	64.8	101.1	0.502
**IL17A, n = 97**	3.9 (3.9–15.8)	17.8	206.1	0.099
**BNP, n = 101**	289 (111–725)	436	807	0.043
**Troponin, n = 94**	37 (22–65)	43	92	0.054
**Galectin, n = 47**	15.8 (12.9–20.5)	17.9	19.8	0.561
**Adiponectin, n = 59**	41.2 (33.8–52.7)	41.9	47.3	0.203
**+ cPCR, 3 samples (%), n = 103**	58 (56.3)	42 (53.2)	16 (66.7)	0.243

*LVEF: Left ventricular ejection fraction. FC: functional class. IFn: Interferon. IL: interleukin. BNP: Brain natriuretic peptide. cPCR–Conventional or Qualitative PCR. qPCR–Quantitative PCR. GDMT: Guideline-directed medical therapy (if > 50% of recommended dose). *CAD Coronary artery disease.

**Table 2:** Multivariable analysis of survival predictor in chagasic patients.

	Univariable analysis		Multivariable analysis	
	HR (IC 95%)	p	HR (IC 95%)	p
Troponin T	1.004 (1.001–1.007)	0.004	1.005 (1.002–1.008)	**0.001**
BNP	1.001 (1.000–1.002)	**<0.001**	1.001 (1.001–1.001)	**<0.001**
Adiponectin	1.029 (1.002–1.057)	0.033	1,042 (1,013–1,072)	0,004
LVEF	0.941 (0.904–0.980)	0.003	0,92 (0.889–0.970)	**<**0.001

**Fig 1:**
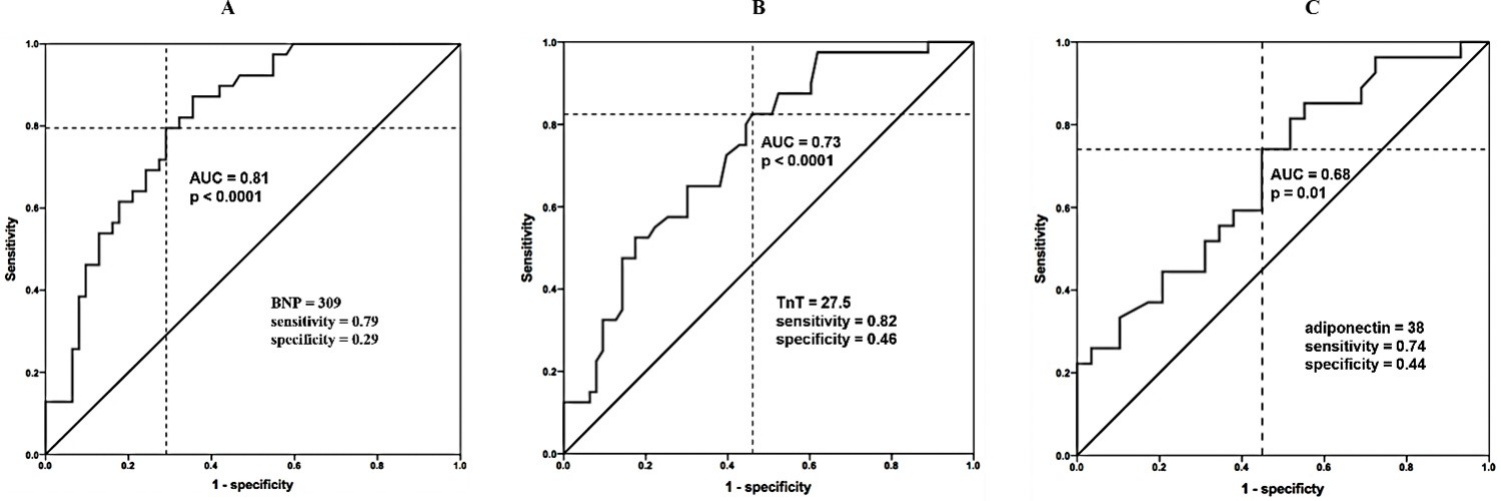
Receiver-operating character (ROC) curves of biomarkers (BNP, troponin T, and adiponectin).

**Fig 2:**
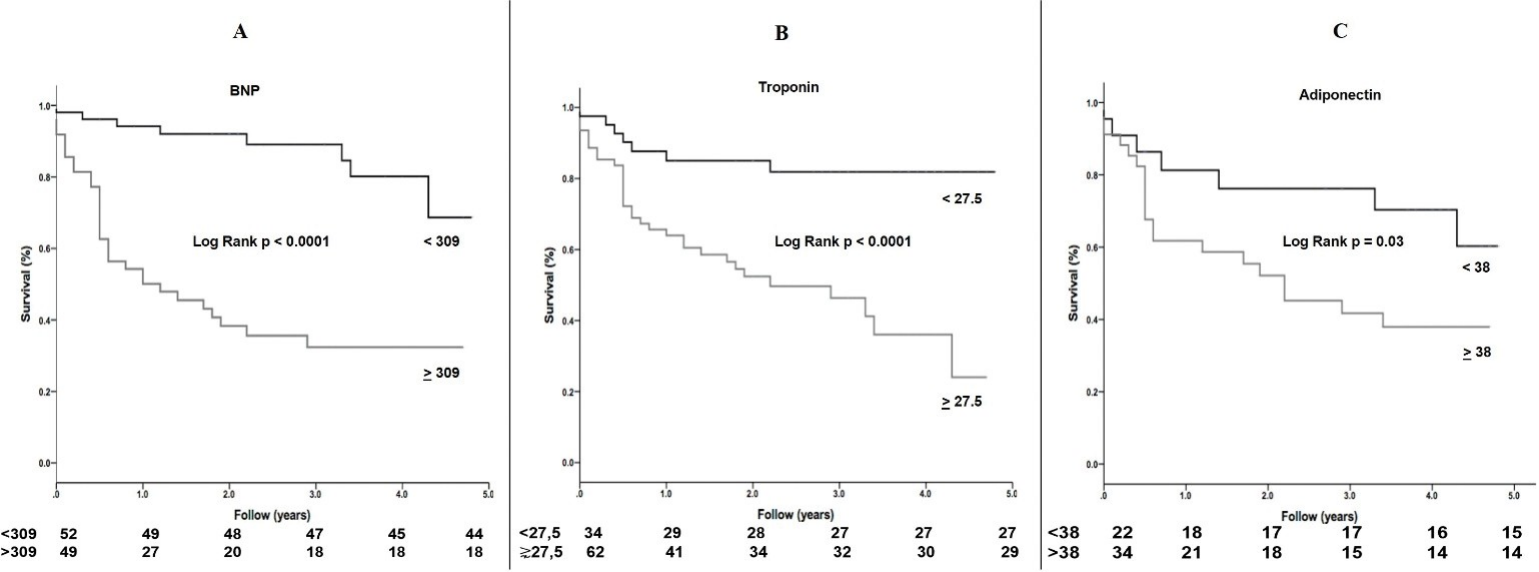
Kaplan-Meier analysis among patients with Chagas’ heart disease and their BNP, TnT e adiponectin values.

**Fig 3:**
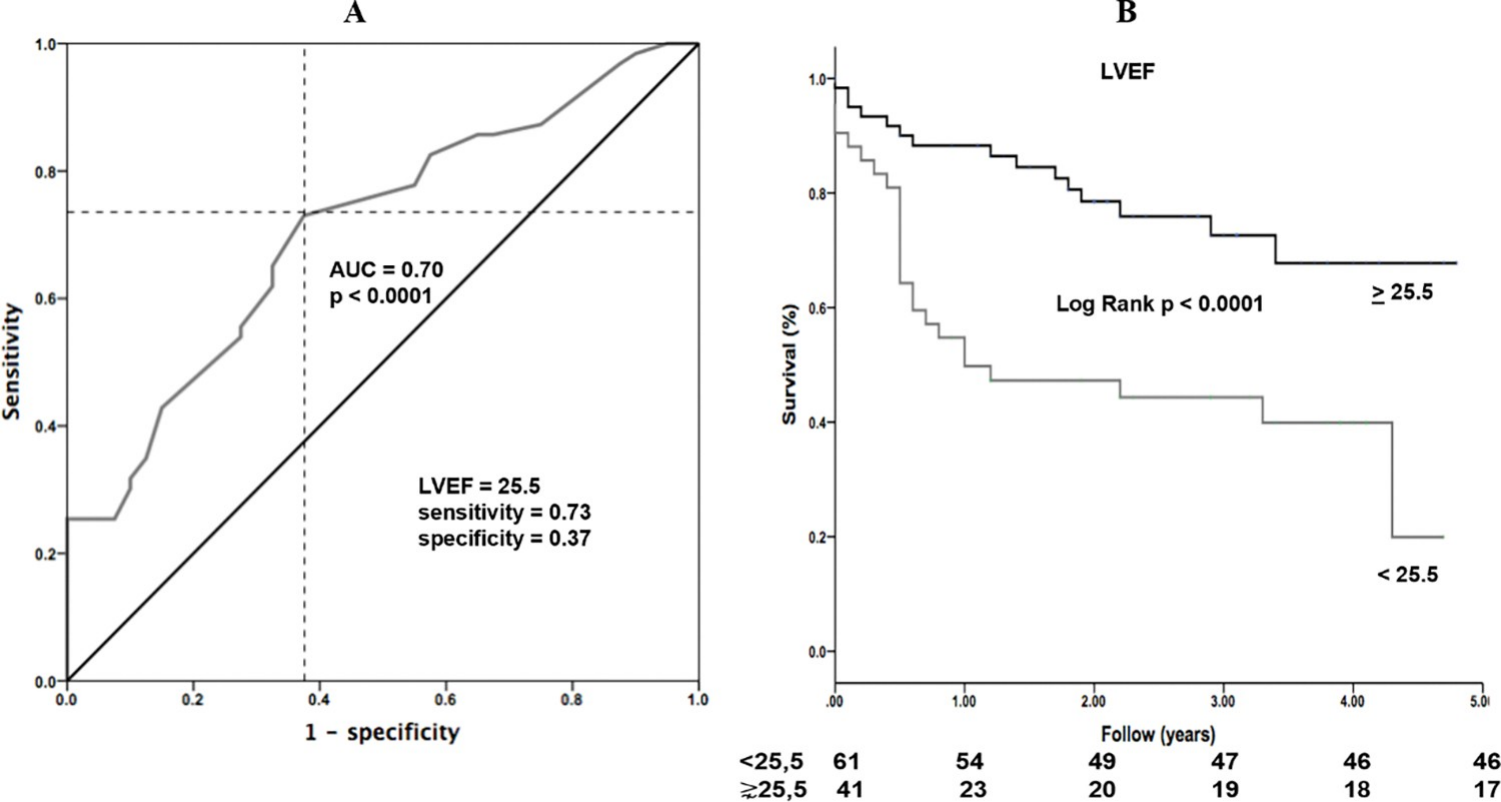
Receiver-operating character (ROC) curves and Kaplan-Meier analysis among patients with Chagas’ heart disease and their LVEF.

The ROC analysis was significant for BNP, TnT, adiponectin, and LVEF (AUC 0.81, p<0.001; AUC 0.73, p<0.001; AUC 0.68, p = 0.01, and AUC 0.70, p < 0.001, respectively) (Fig 1). We identified optional thresholds for BNP of 309 pg/ml, TnT of 27.5 ng/ml, adiponectin of 38 μg/mL, and LVEF of 25.5%. For survival analysis, the threshold value for BNP, TnT, adiponectin, and LVEF was considered for the overall cohort. The median survival estimated by the Kaplan-Meier analysis among patients with Chagas’ heart disease for threshold values of BNP < 309 showed a significant difference when compared to BNP > 309, as well as for TnT < 27.5 and TnT > 27.5, adiponectin between values < 38 and > 38, and LVEF < 25.5 and > 25.5% (Figs 2 and 3). Survival free of mortality from all causes and the need for heart transplantation in 5 years was 49% and mortality from all causes was 62%. The mean survival was 4.1 years (3.8–4.4; 95% CI).

## Discussion

The main findings of the current study showed that the BNP, TnT, and reduced LVEF were identified as strong predictors of lower survival in patients with chagasic HF with HFrEF (thresholds: BNP > 309 pg/ml, TnT > 27.5 ng/ml, LVEF < 25.5%). Besides, the adiponectin levels >38 μg/mL were also found to be a predictor of lower survival, although with a moderate correlation. No evidence was found to support a relationship between inflammatory parameters (including INF-γ, IL-6, IL-10, IL12-(p70), IL17a, and galectin) and lower survival in these patients and the levels of parasitemia did not show a relationship with increased mortality in this study.

Previous studies have demonstrated that elevated biomarkers such as TnT or BNP are associated with worse prognosis in HF, which was confirmed in this study. However, it is important to note that most studies have been conducted in patients with HF of any etiology [13–15]. In the Chagasic population, the association between elevated natriuretic peptide and high-sensitivity TnT with worse prognosis has been corroborated in some studies [16,17]. However, there have been conflicting results, with one study not confirming this relationship when using a non-highly sensitive TnT assay [18,19]. Combining multiple factors for risk stratification appears to be a promising approach. The measurement of natriuretic peptides in combination with biomarkers indicating myocardial necrosis (TnT) and inflammation (high-sensitivity C-reactive protein; hs-CRP) has been shown to improve risk prediction [13,19,20]. Additionally, the presence of ventricular dysfunction, particularly when the ejection fraction is less than 25.5%, has been associated with lower survival and was confirmed in this study [21].

In the present trial, adiponectin, another biomarker involved in the pathophysiology of chagas disease, was found to be elevated and correlated with lower survival, although the correlation was weaker. Similar findings have been reported in other investigations including chagasic patients [22,23]. However, its role in CCM is still controversial. Its high levels are associated with systolic dysfunction, which is probably due to overactivity of adipocytes in patients with HF [24]. This increase could be due to a compensatory anti-inflammatory action [25]. Systemic inflammation has been recognized as a common pathophysiological mechanism in both acute and chronic HF, and it is predictive of poor outcomes regardless of LVEF or New York Heart Association functional class [13,26]. Studies such as ASCEND-HF and TIME-CHF have reported elevated levels of usCR hsCRP in HF patients [26–29].

Among the various inflammatory biomarkers studied in this trial, interleukin-1 (IL-1) was not correlated with decreased survival. However, previous studies have linked IL-1 to impaired systolic and diastolic function through various pathways [28]. Other biomarkers such as IFN-γ, TNF-α, IL-6, and IL-1β have been investigated, and higher levels have been found in patients with Chagasic cardiac disease in other patient cohorts, which contradicts the findings of this current study [28,30,31]. Similarly, elevated expression of IFN-γ has been observed in serum samples from Chagas disease patients with cardiomyopathy compared to those with the indeterminate form [32]. On the other hand, IL-10 expression has been associated with better cardiac function based on left ventricular ejection fraction and left ventricular diastolic diameter values [32,33].

When comparing our cohort with other studies, it is important to note that our study included only patients with CCM and significant ventricular dysfunction, whereas the previously discussed studies had different patient characteristics [31–33]. Our cohort consisted mainly of functional class II and III patients, who were younger and had lower BNP levels. Additionally, most of the other studies were cross-sectional or retrospective, despite having a larger sample size. Therefore, the specific characteristics of our study population, including patients with CCM and significant ventricular dysfunction, may explain the discrepancies in inflammatory biomarker findings compared to other studies [31,34].

The importance of persistent parasitemia in the prognosis of CCM is a topic of discussion. Observational studies have shown that benznidazole treatment, compared to no etiological treatment, is associated with reduced disease progression and negative seroconversion [34,35]. However, once heart disease is established, parasitemia may be less influential in the clinical course, with immunological damage to tissues playing a predominant role [35]. The BENEFIT study, involving 2,854 patients followed for a mean of 5.4 years, demonstrated that benznidazole treatment reduced parasitemia but did not significantly impact prognosis compared to the control group [36].

Another important aspect to highlight is the importance of finding prognostic predictors in this population because risk stratification is essential for the prevention of fatal events [37]. Thus, every Chagas patient must be stratified and is recommended by several clinical guidelines [37]. Ventricular dysfunction, which was a predictor in the current study, is also part of this stratification and some scores [37,38].

There are limited studies correlating inflammatory biomarkers with prognosis in patients with CCM. A study conducted in Venezuela did not find a significant correlation between serum levels of CRP, TNF-α, IL-1β, IL-2, and HF severity [39]. However, other studies have reported a worse prognosis in patients with elevated IL-2 levels and dilated cardiomyopathy [40]. Additionally, elevated levels of IL-6 and CRP have been associated with a poorer prognosis in some studies [41]. Overall, the relationship between inflammatory biomarkers and prognosis in patients with CCM is still not fully understood, and further research is needed to elucidate their role.

It is crucial to mention how the discrete typing units (DTUs) of T. cruzi can influence the pathogenesis of CCM, which are collectively known as six DTUs (TcI—TcVI). Among these, TcI exhibits the widest geographical distribution, encompassing both sylvatic and domestic cycles across North America, Central America, and South America. Infections associated with this DTU often present with severe cardiomyopathies [42]. Conversely, TcII is predominantly found in domestic cycles and is linked to patients with cardiac disorders characterized by megaoesophagus and megacolon. TcIII exhibits low pathogenicity. As of now, there is no definitive evidence suggesting that specific T. cruzi genotypes directly induce a particular clinical outcome in chronic Chagas disease in the absence of other contributing factors. However, it is evident that the distinct clinical presentations of Chagas disease are influenced by both host and parasite factors [42].

## Limitations and strengths

Firstly, it was a single-center study. Secondly, the sample size of patients was relatively small. Thirdly, a substantial number of patients (581) were excluded due to different etiologies, recommendations for heart transplantation, or dropout. Despite these limitations, this trial boasts strengths including its prospective cohort design and the comprehensive array of markers employed, encompassing systemic and cardiac inflammatory host markers, cardiac functional markers, and qualitative and quantitative parasite markers. Another noteworthy limitation is that the kits utilized in this study are more general and may have overlooked patients with milder forms or earlier stages of heart failure (HF).

## Conclusions

In this cohort, the inflammatory biomarkers were not predictors for survival in patients with HFrEF of chagasic etiology. However, strong associations were observed between reduced LVEF, BNP, and TnT levels, and lower survival rates in these patients. These findings suggest that, in chagasic patients with severe HF, the the role of inflammatory markers in predicting survival may be limited.
